# Supplementary material for: Identification of photoperiod-induced specific miRNAs in the adrenal glands of Sunite sheep (Ovis aries)
Source: Front Vet Sci. 2022 Jul 22;9:888207. doi: 10.3389/fvets.2022.888207 (PMC9354845; doi:10.3389/fvets.2022.888207)
Supplement: Supplementary file 1 [file Data_Sheet_1.ZIP › Supplementary file/miRDeep2 file.zip!/miRDeep2 file/Know_Structure/SP42A2/oar-mir-154b.pdf]

miRBase precursor : oar-mir-154b
Total read count : 2028
oar-miR-154b-5p read count : 1890
oar-miR-154b-3p read count : 133
remaining reads : 5

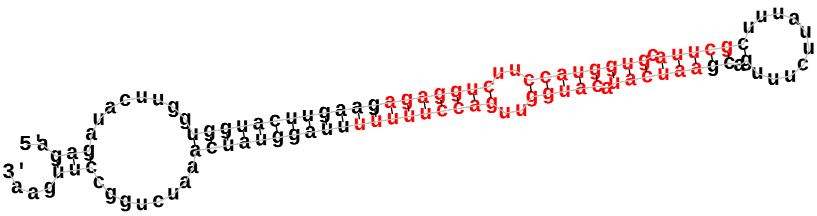

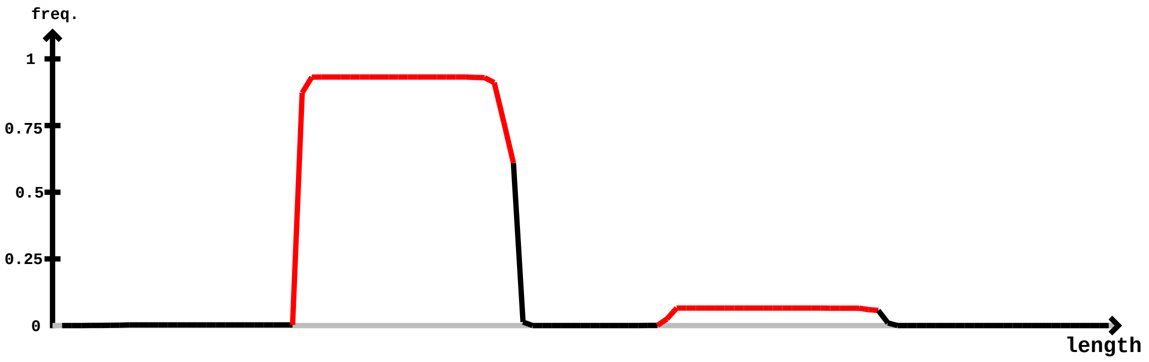


```
                                                                   oar-miR-154b-3p
                                oar-miR-154b-5p
5'- agagauacuuggugguacuugaagagaggucuuccauggugcauucgcuuuauucuuugacgaaucauacaugguugaccuuuuuuuagguaucaaaucuggccuugaa  -3'   exp
    .(((........(((((((((((((((((((..((((((((.((((((..........).)))))))).)))))..)))))))))))))))))))........)))...    reads   mm   sample
    ..agauacuuggugguacuugaag.....................................................................................    1       0    seq
    ....auacuuggugguacuugaag.....................................................................................    1       0    seq
    .....uacuuggugguacuugaag.....................................................................................    1       0    seq
    ......acuuggugguacuugaag.....................................................................................    1       0    seq
    ......acuuggugguacuugaaga....................................................................................    1       0    seq
    ........................agaggucuuccauggugc...................................................................    3       0    seq
    ........................agaggucuuccauggugca..................................................................    2       0    seq
    ........................agaggucuuccauggugcau.................................................................    35      0    seq
    ........................agaggucuuccauggugcauu................................................................    295     0    seq
    ........................agaggucuuccauggugcauuc...............................................................    298     0    seq
    ........................agaggucuuccauggugcauucg..............................................................    1113    0    seq
    ........................agaggucuuccauggugcauucgc.............................................................    23      0    seq
    .........................gaggucuuccauggugcau.................................................................    2       0    seq
    .........................gaggucuuccauggugcauu................................................................    6       0    seq
    .........................gaggucuuccauggugcauuc...............................................................    12      0    seq
    .........................gaggucuuccauggugcauucg..............................................................    96      0    seq
    .........................gaggucuuccauggugcauucgc.............................................................    4       0    seq
    ..........................aggucuuccauggugcauu................................................................    1       0    seq
    ............................................................cgaaucauacaugguugaccuuu..........................    1       0    seq
    ..............................................................aaucauacaugguugaU..............................    1       1    seq
    ..............................................................aaucauacaugguugaccuuu..........................    10      0    seq
    ..............................................................aaucauacaugguugaccuuuu.........................    4       0    seq
    ..............................................................aaucauacaugguugaAcuuuu.........................    1       1    seq
    ..............................................................aaucauacaugguugaccuuuuu........................    29      0    seq
    ..............................................................aaucauacaugguugaccuuuuuu.......................    5       0    seq
    ...............................................................aucauacaugguugaccuuuu.........................    1       0    seq
    ...............................................................aucauacaugguugaccuuuuuu.......................    66      0    seq
    ...............................................................aucauacaugguugaccuuuuuuu......................    15      0    seq
```
